# Substrates Modulate Charge-Reorganization Allosteric Effects in Protein–Protein Association

**DOI:** 10.1021/acs.jpclett.1c00437

**Published:** 2021-03-12

**Authors:** Shirsendu Ghosh, Koyel Banerjee-Ghosh, Dorit Levy, Inbal Riven, Ron Naaman, Gilad Haran

**Affiliations:** Department of Chemical and Biological Physics, Weizmann Institute, Rehovot 76100, Israel

## Abstract

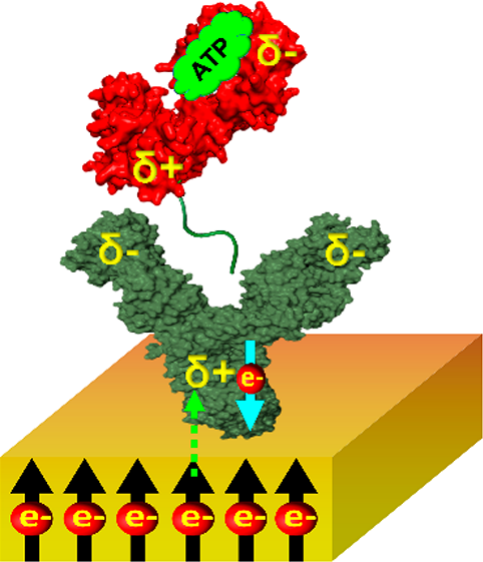

Protein function may be modulated by an event occurring far away from the functional site, a phenomenon termed allostery. While classically allostery involves conformational changes, we recently observed that charge redistribution within an antibody can also lead to an allosteric effect, modulating the kinetics of binding to target antigen. In the present work, we study the association of a polyhistidine tagged enzyme (phosphoglycerate kinase, PGK) to surface-immobilized anti-His antibodies, finding a significant Charge-Reorganization Allostery (CRA) effect. We further observe that PGK’s negatively charged nucleotide substrates modulate CRA substantially, even though they bind far away from the His-tag–antibody interaction interface. In particular, binding of ATP reduces CRA by more than 50%. The results indicate that CRA is affected by the binding of charged molecules to a protein and provide further insight into the significant role that charge redistribution can play in protein function.

An important aspect of protein function and stability is the relative role of local versus nonlocal, long-range, interactions. Allostery, a prime example of a nonlocal effect, is defined as the modulation of the function of a protein by a perturbation that takes place at a site far away from the active site. This phenomenon is essential in biology, as proteins use allostery to control their biological activity.^1,2^ The classical model of allostery invokes a conformational change that accompanies the binding of an allosteric modulator to a protein.^3−5^ However, in our recently published study, we showed that allostery can also be driven by a reorganization of charges within a protein that results from a modulation of its polarizability.^6^ We demonstrated this charge-reorganization allostery (CRA) by studying the effect of charge injection on antibody–antigen association kinetics. In the present study, we discover that a small charged ligand that binds to the antigen may significantly modulate the CRA effect. This new concept of charge reorganization is consistent with the recent discovery of long-range electron conduction within proteins^7^ and the finding of long-range modulation of electric fields in proteins.^8^

We study here a polyhistidine (His)-tagged version of the enzyme phosphoglycerate kinase (PGK) from *Saccharomyces cerevisiae*, Baker’s yeast, as an antigen that binds from solution to a surface-adsorbed anti-His antibody. PGK is an essential enzyme in glycolysis, which generates ADP and 1, 3-diphosphoglycerate from ATP and 3-phosphoglycerate, or vice versa. Nucleotide binding to the enzyme requires a concomitant binding of a magnesium ion.^9^ In our experiment, the His-tag is attached at the C-terminus of PGK and we study the kinetics of its interaction with an anti-His antibody attached to a magnetized metal surface, which serves as an electron source.

Specifically, the antibody is adsorbed on a gold (2 nm)-coated Ni (120 nm) surface using dithiobis[succinimidyl]propionate (DSP) as a linker. The rate of the antigen–antibody association is measured with the substrate magnetized either with its North magnetic pole pointing UP (H+) or DOWN (H−), or with a nonmagnetized substrate. Fluorescently labeled His-tagged PGK molecules are added to the solution on top of the magnetized substrate to initiate binding. At specific time intervals, following the initiation of the association reaction, the substrate is taken out of solution and immediately rinsed. The number of bound antigen molecules is counted using a fluorescent microscope. From Figure 1A,B, it is revealed that the association reaction is faster when the surface is magnetized with the North pole of magnet pointing down (H−). As a control, the antigen–antibody interaction is also studied with a nonmagnetized substrate, which as expected, gives a result that is essentially the average of the two other measurements (Figure 1B).

**Figure 1:**
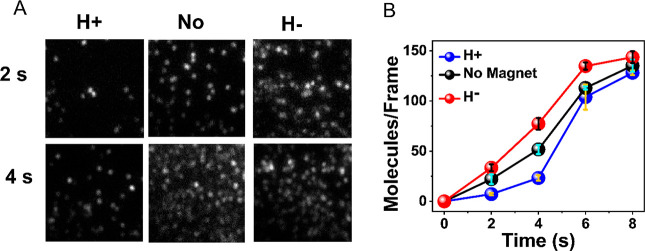
CRA in the binding of His-tagged PGK to an anti-His antibody. (A) Fluorescence images of individual complexes formed between anti-His antibodies adsorbed on a magnetized surface and fluorescently labeled His-tagged PGK molecules. The images are taken following two different interaction periods and with the North pole of the magnetic field pointing either UP (H+) or DOWN (H−) and also in absence of a magnet (No). (B) Antigen–antibody association kinetics under the two magnetic orientations and without the magnet. Error bars represent standard errors of the mean.

These results are similar to our previously reported results, where the antigen used was a His-tagged version of a different protein, ClpB, and can be explained with the same CRA mechanism.^6^ Briefly, when a protein molecule approaches the antibody, it induces charge polarization that results in charge reorganization within the antibody. This charge reorganization depends on the polarizability of the antibody, which is enhanced if electrons can move either from it to the metallic surface or vice versa. Since it is now well established that for chiral molecules charge polarization is accompanied by spin polarization,^10^ facile electron transfer between the antibody and the metallic surface depends on the spin orientation of the electrons in the ferromagnetic substrate, which is modulated by the direction of the magnetic field. This effect is referred to as the chiral induced spin selectivity (CISS) and serves as an effective valve for the flow of electrons from the surface into the adsorbed protein molecules.^11^ It can therefore lead to either acceleration or retardation of antibody–antigen association kinetics, depending on the orientation of the substrate’s magnetization.

In the current case, as a PGK molecule approaches the antibody with its positively charged C-terminus (as observed from a calculation of the dipole moment of the molecule), it polarizes the antibody, inducing a negative charge at the interaction site with the antigen and a positive charge near the surface. Hence, there is an electric potential difference that induces electron flow from the metallic surface into the antibody. However, the rate of this flow depends on the surface magnetization, as discussed above.

We investigated the effect of the binding of negatively charged PGK substrates, Mg-ADP and Mg-ATP, on the CRA effect. As shown in Figure 2A, the CRA effect with ADP is smaller than that shown in Figure 1. ATP suppresses CRA even more, almost eliminating it (Figure 2B). To show this more quantitatively, we compute the selectivity function (*N*_H__–_ – *N*_H__+_) /(*N*_H__–_ + *N*_H__+_), with *N*_H__+_ the number of bound PGK molecules with the magnetic field either pointing up or down. Figure 2C shows the selectivity functions for the three measurements. Since CRA affects the kinetics of protein–protein association, the selectivity is largest at the earliest time point and decays as time progresses. However, the selectivity is clearly reduced in the presence of ADP and ATP. In fact, ATP reduces the selectivity to less than half of its value without the substrates.

**Figure 2:**
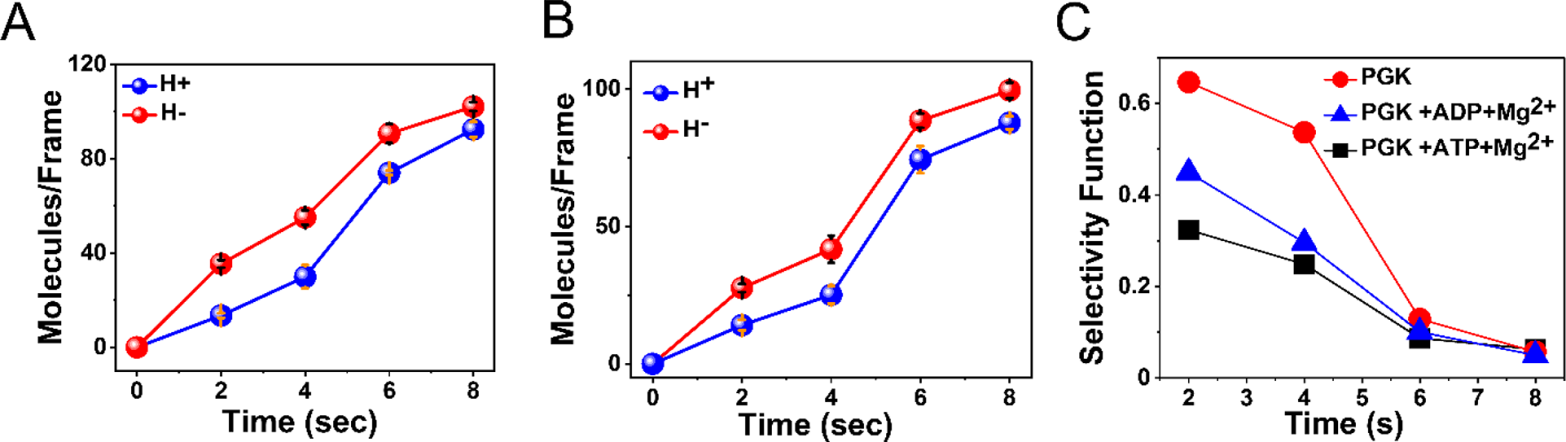
Substrate binding to PGK modulates the CRA effect. (A) Antigen–antibody association kinetics in the presence of Mg-ADP. (B) Antigen–antibody association kinetics in the presence of Mg-ATP. (C) Selectivity function of association kinetics with and without substrates. Error bars represent standard errors of the mean.

To interpret these results, we turn to the cartoons in Figure 3. As already noted, in the absence of substrates the approach of the antigen leads to polarization of the antibody (Figure 3A,B), which in turn leads to charge flow to or from the surface, further enhancing the CRA effect and increasing the rate of the interaction between the proteins. (For a detailed discussion of the role of the electron-flow valve induced by the CISS effect, see Supporting Figure 1.) When a negatively charged substrate, such as ATP, binds to PGK (Figure 3C), there are two potential mechanisms that may operate in parallel and lead to a change in the polarization of the PGK molecule.

**Figure 3:**
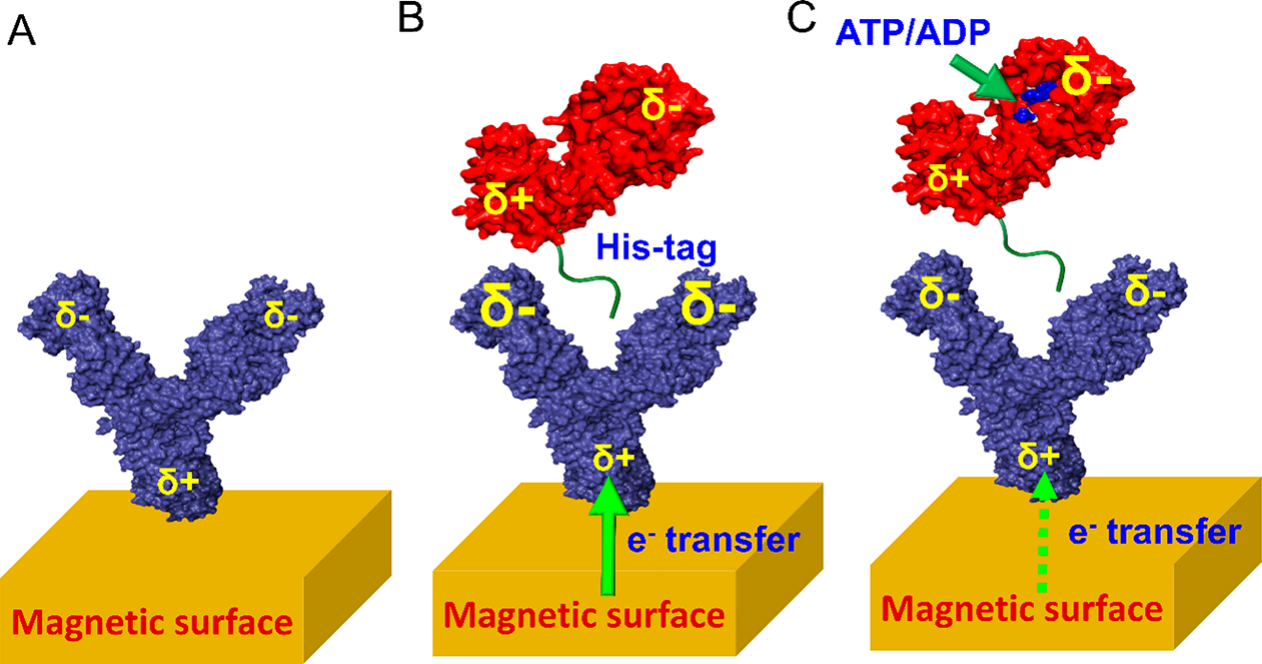
Representation of the events accompanying antigen–antibody interaction. (A) An antibody molecule attached to the surface has a certain distribution of charges, depicted here schematically. (B) Approach of a PGK molecule polarizes the antibody, leading to accumulation of more negative charge close to the binding site, and accordingly to a flow of electrons from the surface into the molecule. (C) When a negatively charged nucleotide is bound to PGK, the protein’s dipole is reduced in size, and hence it does not polarize the antibody to the same extent as in (B), and the electron flow from the surface is slower. The relative size of each δ sign indicates the amount of charge accumulated at a given electric pole.

First, the binding of the ATP moiety may lead to subtle changes in the positions of charged groups within the protein, driven by reorientations of residues and their side chains. Sato et al.^12^ investigated this effect, which they termed “dielectric allostery”, using molecular dynamics simulations of the binding of ATP to myosin. In the case of PGK, this would lead to a reduction of the overall dipole moment of the protein, therefore decreasing the polarization of the antibody by the approaching antigen, and in turn reducing the effect of charge flow from the surface on antibody–antigen association. In addition to this scenario, which is based on the orientational polarizability of PGK, there is another potential mechanism, based on the electronic polarizability of the protein. The binding of ATP may lead to electron transfer from the charged molecule to the protein. This electron transfer event might involve a full electron or only a partial charge, but in any case, it would also affect the overall dipole moment of the protein and thereby modulate its influence on the antibody as it approaches it, with a similar overall result. Therefore, both scenarios discussed here would lead to a modulation of the dipole moment of PGK and in turn to a decrease in the polarization of the antibody and a weaker effect on the association rate due to charge flow from the surface (Figure 3C), as demonstrated experimentally in Figure 2.

This study of antigen–antibody association kinetics, in which the antibody molecules are attached to a ferromagnetic substrate and the antigen is the His-tagged version of the protein PGK, confirms and generalizes the CRA mechanism reported recently.^6^ We further find here that the presence of negatively charged substrate, bound to the antigen molecules, reduces the polarization of the antibody upon binding and in turn reduces the amount of charge transferred from the metallic surface. These observations indicate the presence of charge reorganization in the proteins studied here over long distances. Further, the binding of small charged molecules to one of the proteins induces a CRA effect that significantly modulates their association, even though these small molecules bind at a site that is far away from their interaction region.

In recent years there is a growing interest in the effect of electric fields on protein structure and function,^13−15^ including their role in enzyme catalysis.^16,17^ CRA presents a novel mechanism by which electric fields can have a long-range effect. The physiological relevance of the CRA mechanism remains to be established. We note, however, that molecular dynamics calculations by Takano and co-workers demonstrated that dielectric rearrangements following the binding of ATP to myosin cause an extension of its lever arm region, an important step in its activity as a motor protein.^12,18^ It is therefore likely that the functional impact of the binding of small charged ligands to proteins involves CRA. In future studies we therefore hope to investigate the effect of CRA on essential protein functions, such as enzymatic activity.

## Supporting Information Available

Experimental details and schematic representation of the role of the CISS effect in the proposed explanation for the experimental results (Supporting Figure 1) (PDF)
