# Supplementary material for: Substrates Modulate Charge-Reorganization Allosteric Effects in Protein–Protein Association
Source: J Phys Chem Lett. 2021 Mar 12;12(11):2805–8. doi: 10.1021/acs.jpclett.1c00437 (PMC8041378; doi:10.1021/acs.jpclett.1c00437)
Supplement: Supplementary file 1 — jz1c00437_si_001.pdf [file jz1c00437_si_001.pdf]

Supporting Information

Substrates Modulate Charge-Reorganization Allosteric Effects in Protein-Protein Association

Shirsendu Ghosh,[#] Koyel Banerjee-Ghosh,[#] Dorit Levy, Inbal Riven,
Ron Naaman,* Gilad Haran*
Department of Chemical and Biological Physics, Weizmann Institute,
Rehovot 76100, Israel
E-mail: gilad.haran@weizmann.ac.il; ron.naaman@weizmann.ac.il

Experimental details

Expression, purification and labelling of PGK

Phosphoglycerate kinase (PGK) DNA was cloned into a pET28b vector, fused to a C-terminal 6xHis tag derived from the vector. For site-specific labeling of PGK, the wild-type cysteine at position 97 was replaced by a serine, and a new cysteine residue was incorporated using site-directed mutagenesis, resulting in the C97S S290C PGK mutant.

The C97S S290C PGK plasmid was transformed into *E. Coli* BL21 pLysS (DE3) cells (Invitrogen), and grown in LB media at 37 °C, up to an optical density of 0.8. Protein expression was induced by the addition of 1mM IPTG, and cells were then incubated at 25 °C for overnight. Following expression, bacteria were harvested and the proteins were purified on a Ni-NTA resin, according to the manufacturer's instructions (GE Healthcare). Protein was kept until used at -80 °C in storage buffer (20 mM sodium phosphate, 1 mM TCEP, pH 6.8).

PGK was tagged after buffer exchange into labeling buffer (50 mM Tris, 25mM KCl, pH 7.1), using a desalting column (Sephadex G25, GE Healthcare). Protein was then mixed

with Alexa 647 C2 maleimide (Invitrogen) at a 1:1.5 protein-to-dye ratio for 2h at RT. Labeled molecules were then separated from excess dye using a desalting column.

Kinetic study of the interaction between His-tagged PGK and anti-His antibodies

Ultra-LEAF™ Purified anti-His antibody was attached to the gold coated magnetic surface using dithiobis[succinimidyl] propionate (DSP) as a linker. Surface preparation and immobilization of the antibody were done as in our previous work.[1] A DSP monolayer was formed on the gold surfaces by incubating them in a solution of DSP in DMSO (4 mg/ml) for 30 mins. After that, the surfaces were rinsed with DMSO and water and were incubated into the antibody solution in PBS (1 mg /ml) for 4 h. After rinsing the antibody-immobilized gold surfaces with PBS (pH=7.1), they were kept at 50 mM Tris buffer solution. Then, they were immersed in a solution of PGK (0.05 µM) in 50 mM tris buffer in a MAKTEK glass bottom petri-dish kept on a permanent magnet for different time intervals (2 s, 4 s, 6 s, 8 s) and immediately taken out and rinsed with buffer. The reaction kinetics were studied with both orientations (either H+ or H-) of the magnet and also in absence of the magnetic field as a control. The interaction was also studied in the presence of ADP (2.5 mM) or ATP (2.5 mM) in a magnesium chloride (3 mM) solution. Fluorescence imaging was carried out immediately following sample preparation. All samples were prepared twice to test reproducibility of the results.

Microscopy experiments & data analysis

The fluorescence imaging of the samples was done using a home-built total internal reflection fluorescence microscope (TIRFM). A detailed description of the TIRFM setup is given elsewhere.[2] We recorded the data and analyzed them following the same procedure as reported in the Ref 1. In each experiment, 10 different TIRFM movies were recorded on

10 different regions with a size of 101 pixel X 101 pixel, i.e. 6.73μm X 6.73 μm of the sample. On each region, we recorded 100 ms frames until all molecules in the designated area were photo bleached. TIRFM movies were analyzed using custom-written Matlab (MathWorks) routines. Individual spots were identified in the first frame of a movie using a combination of thresholding and center of mass (CM) analysis as described previously.[3]

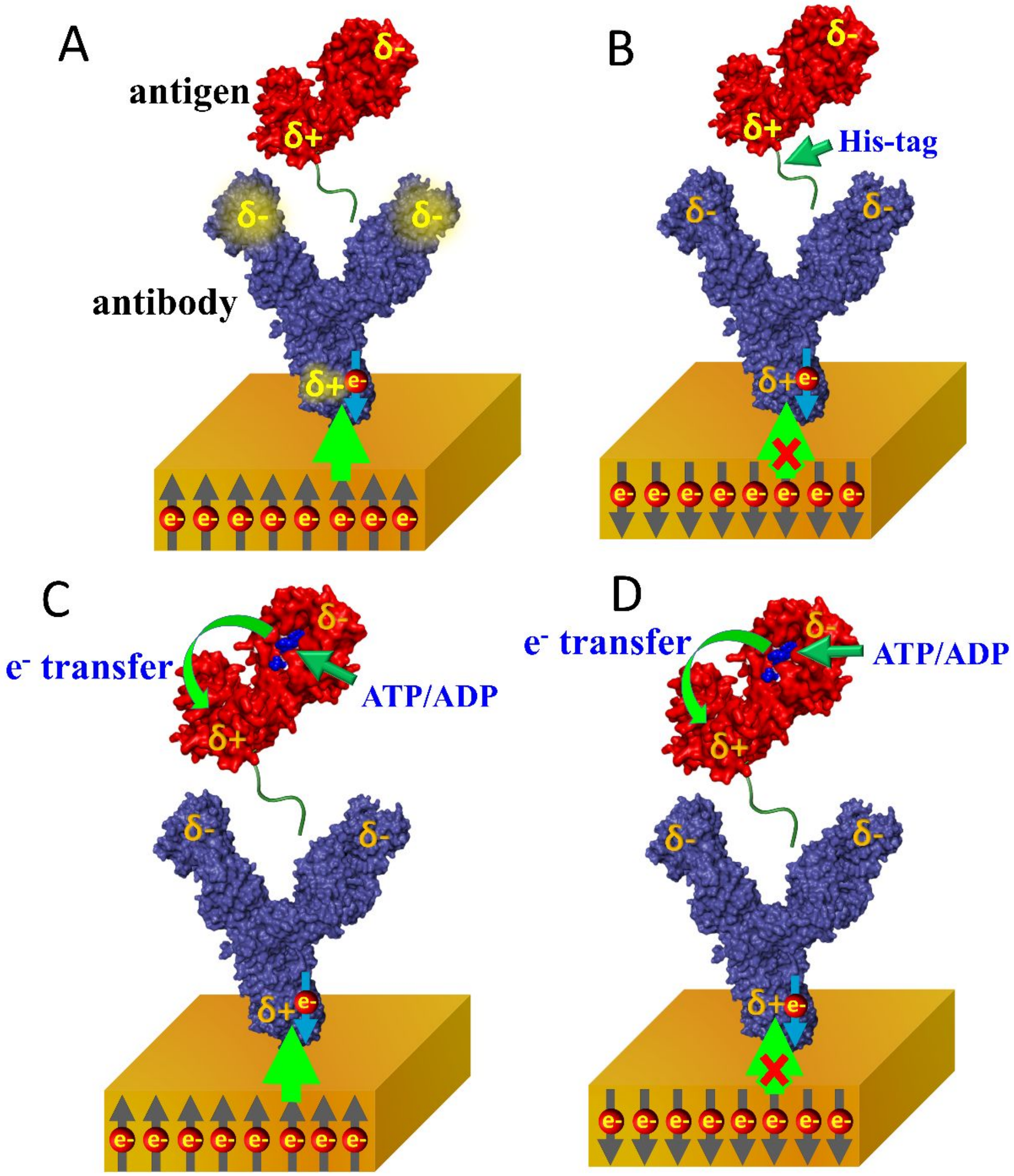

Supporting figure 1: Schematic representation of the role of the CISS effect in the proposed explanation for the experimental results. (A) as in our previous study, one direction of substrate magnetization facilitates electron transfer from metal substrate to the antibody following polarization of the latter by the approaching antigen; the result is a faster protein-protein association process. (B) When the substrate is magnetized in the opposite direction, charge flow into the antibody is hindered and results in an inefficient CRA in the antibody and a slow association reaction. (C-D) In the presence of ADP or ATP, changes in the polarization of the antigen lead to less positive charge close to its C

terminus and therefore less electrons are attracted from the substrate, either when electron transfer is allowed (C) or not (D).

References

(1) Banerjee-Ghosh, K.; Ghosh, S.; Mazal, H.; Riven, I.; Haran, G.; Naaman. R. Long-range charge reorganization as an allosteric control signal in proteins. *J. Am. Chem. Soc***. 2020,** *142***,** 20456–20462.

(2) Jung, Y.; Riven, I.; Feigelson, S. W.; Kartvelishvily, E.; Tohya, K.; Miyasaka, M.; Alon, R.; Haran, G. Three-dimensional localization of T-cell receptors in relation to microvilli using a combination of superresolution microscopies. *Proc. Natl. Acad. Sci. U. S. A.* **2016***, 113*, E5916-E5924.

(3) Henriques*,* R.; Lelek, M.; Fornasiero, E. F.; Valtorta, F.; Zimmer, C.; Mhlanga, M. M. QuickPALM:3D real-time photoactivation nanoscopy image processing in Image J, *Nat. Methods* **2010**, *7*, 339-340.
